# Antibiofilm Activity of *Weissella* spp. and *Bacillus coagulans* Isolated from Equine Skin against *Staphylococcus aureus*

**DOI:** 10.3390/life12122135

**Published:** 2022-12-17

**Authors:** Eva Styková, Radomíra Nemcová, Marián Maďar, Dobroslava Bujňáková, Rastislav Mucha, Soňa Gancarčíková, Francisco Requena Domenech

**Affiliations:** 1 Clinic of Horses, University Veterinary Hospital, University of Veterinary Medicine and Pharmacy in Košice, Komenského 73, 041 81 Košice, Slovakia; 2 Department of Microbiology and Immunology, University of Veterinary Medicine and Pharmacy in Košice, Komenského 73, 041 81 Košice, Slovakia; 3 Institute of Animal Physiology, Centre of Biosciences of the Slovak Academy of Sciences, Šoltésovej 4, 040 01 Košice, Slovakia; 4 Institute of Neurobiology, Biomedical Research Center of the Slovak Academy of Sciences, Šoltésovej 4, 040 01 Košice, Slovakia; 5 Department of Cell Biology, Physiology and Immunology, University of Córdoba, 14014 Córdoba, Spain

**Keywords:** antibiofilm activity, antimicrobial activity, *Bacillus coagulans*, probiotic, skin, *Weissella* spp.

## Abstract

The aim of this study was to evaluate the antimicrobial and antibiofilm activity of *Weissella cibaria*, *Weissella hellenica* and *Bacillus coagulans*, isolated from equine skin, against biofilm-forming *Staphylococcus aureus* CCM 4223 and clinical isolate methicillin-resistant *S. aureus* (MRSA). Non-neutralized cell-free supernatants (nnCFS) of tested skin isolates completely inhibited the growth and biofilm formation of *S. aureus* strains and caused dispersion of the 24 h preformed biofilm in the range of 21–90%. The majority of the pH-neutralized cell-free supernatants (nCFS) of skin isolates inhibited the biofilm formation of both *S. aureus* strains in the range of 20–100%. The dispersion activity of *B. coagulans* nCFS ranged from 17 to 77% and was significantly lower than that of nnCFS, except for *B. coagulans* 3T27 against *S. aureus* CCM 4223. Changes in the growth of *S. aureus* CCM 4223 in the presence of catalase- or trypsin-treated *W. hellenica* 4/2D23 and *W. cibaria* 4/8D37 nCFS indicated the role of peroxides and/or bacteriocin in their antimicrobial activities. For the first time, the presence of the *fenD* gene, associated with biosurfactants production, was detected in *B. coagulans*. The results of this study showed that selected isolates may have the potential for the prevention and treatment of biofilm-forming *S. aureus* infections.

## 1. Introduction

Most microbial skin infections are based on the formation of biofilms, which are an important reservoir of pathogens. Biofilm-forming microorganisms are characterized by an increased resistance to antimicrobial, antifungal and disinfectant agents and the ability to resist the host’s immune system. The antibiotic therapy of biofilm infections is very demanding and often insufficient, so these infections have a long-term, often reversible character [1]. On a global scale, there is an effort to find new approaches and to use targeted and rational strategies to address this issue without the risk of inducing resistance [2,3]. The methods based on beneficial bacteria and their bioactive compounds are becoming more and more current and sought after [4,5,6]. The potential use of probiotic strains capable of producing potent antimicrobial agents, i.e., bacteriocins, hydrogen peroxide, organic acids, exopolysaccharides and biosurfactants (BS), has received increasing attention in terms of the successful prevention of pathogen adhesion [7].

*Weissella* spp. and *Bacillus coagulans* are found in a wide variety of ecological niches, from humans to fermented foods [8,9]. They have been found to act as probiotics, mainly due to their antimicrobial activity, as is the case with certain bacteriocinogenic strains of *Weissella paramesenteroides*, *Weissella hellenica* and *Weissella cibaria* [9]. In some strains of *B. coagulans*, there have been bacteriocins with a broad spectrum of inhibitory activity against *Escherichia coli*, *Pseudomonas aeruginosa*, *Klebsiella pneumoniae*, *Bacillus cereus*, *S. aureus*, vancomycin-resistant enterococci as well as fungi and yeasts [10,11]. *B. coagulans* could be used for topical skin and surface applications for the purpose of the reduction or prevention of microbial infections [12].

*Weissella cibaria* CMU has been known to produce high amounts of hydrogen peroxide, which strongly inhibited the biofilm formation of *Streptococcus mutans* [13,14]. Most of the antibacterial effects of *W. cibaria* CMU were found to be acid- and hydrogen peroxide-dose-dependent. Furthermore, the proteomic analysis of the *W. cibaria* CMU secretome identified *N*-acetylmuramidase as a potential anti-microbial agent that is effective against *P. gingivalis* [15]. Biosurfactants produced by immobilized *W. cibaria* PN3 cells at concentrations ranging from 10 to 16 mg/mL showed antimicrobial activity toward *S. aureus*, *E. coli* and *Candida albicans* [16].

Equine pastern dermatitis is considered a syndrome of the specific cutaneous reaction patterns caused by various inflammatory skin conditions, in which mixed infections such as bacterial, fungal, yeast or scabies infections are involved [17]. Secondary bacterial infections with *Staphylococcus* spp. are a common issue that often complicates the diagnosis [18]. In a study by Kaiser-Thom et al. [19], they focused on the skin microbiota of horses with equine pastern dermatitis; four different numerous families (*Staphylococcaceae*, *Sphingomonadaceae*, *Burkholderiaceae*, *Microbacteriaceae*) were detected by the next-generation sequencing (NGS) method, of which *Staphylococcaceae* had the highest representation. This family includes the well-known genus *Staphylococcus*, several species of which are considered opportunistic pathogens [20]. Kaiser-Thom et al. [19] found that pasterns affected by dermatitis developed a bacterial imbalance in favor of staphylococcal species. The growth of staphylococci is a frequently reported phenomenon after the disruption of the skin barrier not only in humans [21] but also in horses [22]. For example, a study by Chiers et al. [23] detected staphylococci in almost a third of the samples from horses with skin diseases of various etiologies. Similarly, a large German survey of wound infection rates in companion animals found “alarming rates of MRSA (methicillin-resistant *Staphylococcus aureus*)”, and 23% of the horse samples tested were positive for *S. aureus* [24].

This study focused on the isolation of the potentially beneficial bacteria *Weissella* spp. and *B. coagulans* from the healthy skin of distal parts of equine limbs and on the in vitro screening of their ability to prevent biofilm formation and to disperse the preformed biofilms of the pathogenic strains *Staphylococcus aureus* CCM 4223 and methicillin-resistant *S. aureus* (MRSA).

## 2. Materials and Methods

### 2.1. Isolation of Microorganisms

Skin swabs were collected from the distal part of the limbs and other regions (pastern, the middle line of the chest, the pectoral area) of the body of 15 clinically healthy broodmares of Norik breed Muráň Plain type. The horses were kept on pasture in the Center of Horse breeding in Dobšiná, Slovak Republic. The swabs were placed into 1 mL of isotonic saline solution, and a series of 10-fold dilutions was prepared. From the appropriate dilutions, 0.1 mL aliquots were spread onto de Man-Rogosa-Sharpe agar (MRS, pH 6.4; Carl Roth GmbH + Co., KG, Karlsruhe, Germany) and cultivated under aerobic and anaerobic conditions (Gas Pak Plus, BBL, Microbiology Systems, Cockeysville, MD, USA) at 27 °C for 48–72 h. The morphologically different colonies were reclaimed from the individual dilutions under the same growth conditions to obtain pure cultures. The isolates were selected based on gram-positive staining and coccoid or rod shapes. The selected isolates were preserved in Microbank^TM^ (Pro-Lab Diagnostics, Richmond Hill, ON, Canada) at −70 °C.

### 2.2. Hemolytic Activity Test

The isolates were spread onto Trypticase soy agar (TSA; pH 6.9; Oxoid Unipath, Ltd., Basingstoke, UK) with 10% of horse blood. The plates were incubated under the above-mentioned conditions, which were optimal for each strain. The positivity of isolates was indicated by the lysis of blood cells and a colorless transparent ring around the colonies.

### 2.3. Genotypic Identification of the Isolates

The isolation of DNA from non-hemolytic isolates was performed by DNAzol direct (Molecular Research Center Inc., Cincinnati, OH, USA) according to the manufacturer’s instructions. The 16S ribosomal RNA (rRNA) genes from the isolates were amplified by PCR using the universal primers [25] Bac27F (5-AGAGTTTGATCMTGGCTCAG-3) and 1492R (5-CGGYTACCTTGTTACGACTT-3). The PCR reactions were carried out in a total mastermix, with a 50 μL volume per sample, containing 2 μL of the extracted DNA and 46 μL of a reaction mixture comprising One Taq 2X Master Mix with Standard Buffer (New England Biolabs, Hitchin, UK), diluted with water for molecular biology (PanReac AppliChem, Darmstadt, Germany) to a 1X concentration, and 1 μL of each primer was added at a concentration of 33 μM. The PCR cycling conditions were as follows: initial denaturation at 94 °C for 5 min, followed by 30 cycles of denaturation at 94 °C for 1 min; annealing at 55 °C for 1 min; primer extension at 72 °C for 3 min; and, finally, a primer extension at 72 °C for 10 min. The PCR was run on a thermocycler (TProfesional Basic, Biometra GmbH, Göttingen, Germany), aliquot PCR products were separated by horizontal 3% (*w*/*v*) agarose gel electrophoresis in Tris-acetate-EDTA buffer (pH 7.8) and visualized with GelRed (Biotium, Inc., Hayward, CA, USA) under ultraviolet light. The positive samples were selected for sequencing. Regarding samples in which the product was not present, a different extraction kit was used. DNA was extracted by the Quick-DNA Fecal/Soil Microbe Miniprep Kit (Zymo Research, Irvine, CA, USA) according to the manufacturer’s instructions. After that, the purity and concentration of the DNA isolated by the kit were measured using a NanoDrop™ 8000 Spectrophotometer (Thermo Fisher Scientific, Gloucester, UK). The PCR conditions were the same as those in the case of the DNAzol direct isolation protocol, except for the set-up volume and concentration of the DNA optimal for routine PCR. The amplification products were sent to Microsynth Austria GmbH (Vienna, Austria) for sequencing in both directions using 1492R and Bac27F primers. The obtained sequences were validated and assembled by Geneious 8.0.5 (Biomatters, Auckland, New Zealand). The bacterial species were identified based on the consensus sequence of the 16S rRNA gene and by genotyping using online BLASTn analysis (https://BLAST.ncbi.nlm.nih.gov/BLAST.cgi, accessed on 20 September 2018).

The 16S rRNA genes of these bacteria were submitted to the GenBank database and published under the following accession numbers: *Bacillus coagulans* 3T27 (AN: MN559542), *Bacillus coagulans* 9FT27 (AN: MN559543), *Weissella cibaria* 4/8D37 (AN: MN559544), *Weissella hellenica* 1/7D23 (AN: MN559545), *Weissella hellenica* 4/2D23 (AN: MN559546), *Weissella hellenica* 7/1D23 (AN: MN559547).

### 2.4. Antibiotic Susceptibility Testing and MIC Determinations

Antibiotic susceptibility testing was performed according to the ISO 10932/IDF 223 standard [26]. The minimum inhibitory concentrations (MICs) of the genotypically identified strains of *Weissella* spp. and *B. coagulans* towards 16 antibiotics, namely, gentamicin, kanamycin, streptomycin, neomycin, tetracycline, erythromycin, clindamycin, chloramphenicol, ampicillin, penicillin, vancomycin, virginiamycin, linezolid, trimethoprim, ciprofloxacin and rifampicin, were determined by the microdilution method using the microtiter VetMIC Lact-1 and Lact-2 panels for the susceptibility testing of bacteria (Statens Veterinärmedicinska Anstalt, Uppsala, Sweden). Briefly, individual *Weissella* spp. and *B. coagulans* colonies grown on MRS or Mueller–Hinton (MH) agar (Oxoid, Basingstoke, Hampshire, UK) were resuspended in 2 mL of sterile saline solution (Oxoid, England) to obtain a density corresponding to 1 McFarland (approximately 3 × 10^8^ CFU/mL). This suspension was diluted 1000-fold in LSM medium (lactic acid bacteria susceptibility test medium) (consisting of 90% IST (Iso-Sensitest) broth (Oxoid, England) and 10% MRS broth (Oxoid, England)) for *Weissella* spp. and in IST medium for *B. coagulans.* Then, 100 µL (approximately 3 × 10^4^ CFU/well) of the dilution was added to each well of the microdilution plate, and the plates were incubated at 30 °C for 48 h and at 35 °C for 24 h under anaerobic and aerobic conditions, respectively. The growth within each well was examined visually after incubation for each antibiotic and compared with the positive control, as recommended by the ISO 10932/IDF 223 standard. The MICs (µg/mL) were interpreted according to the recent FEEDAP document of the European Food Safety Authority (EFSA) on the update of the criteria used in the assessment of antibiotic bacterial resistance of human or veterinary importance [27,28], as well as the epidemiological cut-off values defined by the ACE e ART Project results, ISO 10932 [26].

### 2.5. Preparation of the Cell-Free Supernatant (CFS) of Skin Isolates

The CFS of skin isolates was prepared according to Lin et al. [29], with some modifications. Briefly, *Weissella* spp. were inoculated onto MRS agar (pH 6.4; Carl Roth GmbH + CO., KG) and incubated under anaerobic conditions (GasPak Plus, BBL) at 37 °C for 72 h. *B. coagulans* were inoculated onto Brain Heart Infusion (BHI) agar (pH 7; HiMedia Laboratories, Mumbai, India) and incubated under aerobic conditions at 37 °C for 48 h. For the preparation of a standardized suspension, 3–4 solitary colonies were resuspended in 5 mL of saline solution and adjusted to the density of 1 McFarland. Then, 0.5 mL of each suspension was inoculated into 50 mL of MRS broth without Tween (MRS-T; bacteriological peptone 10 g/L (HiMedia Laboratories, Mumbai, India), beef extract 8 g/L (Acumedia and Lab M, Lansing, MI, USA), yeast extract 5 g/L (Laboratorios Conda S. A., Madrid, Spain), glucose 20 g/L (Slavus Ltd., Bratislava, Slovak Republic), sodium acetate 3 g/L (99%; Lachema, Brno, Czech Republic), K_2_HPO_4_ 2 g/L (99%; Centralchem Ltd., Bratislava, Slovak Republic), triammonium citrate 2 g/L (99%; Fisher Scientific, Leeds, UK), MgSO_4_ 0.2 g/L (99,5%; Centralchem Ltd., Bratislava, Slovak Republic) and MnSO_4_ 0.05 g/L (99%; Carl Roth GmbH + CO., KG), all in distilled water, pH 7) and incubated in a water bath with shaking (LSB Aqua Pro, Grant Instruments, Cambridge, UK) at 119 rpm and 37 °C for 48 h. After cultivation, the cultures were centrifuged at 4173× *g* and 4 °C for 40 min. The supernatant of each skin isolate was divided into two equal parts. One part was left non-neutralized (nnCFS with a pH in the range of 3.4–4.5), and the second one was neutralized (nCFS pH 7), with 10 M NaOH used to eliminate the effect of organic acids. Both parts of the CFS were filtered through a syringe filter (0.22 µm, Fisher Scientific Ltd., Dublin, Ireland) and stored at −70 °C.

### 2.6. Organic Acids Production Analysis

An analysis of the lactic, acetic, formic, succinic and acetoacetic acids in the nnCFS of *Weissella* spp. and *B. coagulans* was conducted by capillary isotachophoresis, as described previously by Styková et al. [30]. The measurements were carried out using an Isotachophoretic analyzer ZKI 01 (Radioecological Institute, Košice, Slovak Republic). The testing was carried out in triplicate, and the measured levels of organic acids were expressed as mM/L ± SD.

### 2.7. Effect of the CFS of the Skin Isolates on the Growth and Biofilm Formation of Staphylococcus aureus CCM 4223 and MRSA

A pathogenic strain of *S. aureus* CCM 4223 (Czech Collection of Microorganisms, Brno) isolated from the wound and our clinical isolate MRSA obtained during arthroscopy from the infected equine pastern joint were used in the study. These strains were characterized by the presence of the *icaADCB*, *agrA*, *srtA* and i*caR* operon genes responsible for biofilm formation [31]. MRSA was identified in LABOKLIN (Laboratory for Clinical Diagnostics GmbH & Co., KG, Bad Kissingen, Germany), the presence of the *mec*A gene was confirmed in Microsynth (Wien, Austria). The strain was published in the GenBank database as *Staphylococcus aureus* MRSA (AN: MN822720). Pathogenic strains were grown overnight at 37 °C on BHI agar (pH 7; HiMedia Laboratories). For the preparation of a standardized suspension of *S. aureus*, three to four solitary colonies were resuspended in 5 mL of saline solution and adjusted to the density of one McFarland (approximately 3 × 10^8^ CFU/mL). An aliquot (25 µL) of this suspension was added to 2.5 mL of MRS-T medium (pH = 7) and to 2.5 mL of nnCFS or nCFS. As a negative control, individual media without bacterial culture were used, which were treated in the same way. Subsequently, a 200 µL sample was pipetted to the wells of 96-well microtiter plates (Greiner ELISA 8 Well Strips, 350 µL, Flat Bottom, Medium Binding; Cruinn Diagnostics Ltd., Dublin, Ireland). MRS-T medium with a pathogenic strain was used as a positive control regarding the growth of this bacterium. The microtiter plates were incubated aerobically at 37 °C for 24 h. After incubation, the growth of *S. aureus* was measured by a Synergy 4 Multi-Mode microplate reader (BioTek Instruments Inc., Winooski, VT, USA) at a 570 nm wavelength. The results were expressed as the percentage of growth inhibition

% growth inhibition = [1 − (A_CFS_/Ao)] × 100

where A_CFS_ is the absorbance of the well content with CFS and Ao is the absorbance of the control well.

The biofilm formation was determined using a modified crystal violet assay according to O’ Toole et al. [32]. Briefly, the supernatant was removed, and the wells were washed three times with deionized water and allowed to dry at room temperature for 40 min. After drying, the wells were stained with 200 µL of a 0.1% crystal violet solution (Merck Ltd., Praha, Czech Republic) and incubated at room temperature for 30 min. Subsequently, the excess color was removed, and the wells were washed three times with deionized water and allowed to dry at room temperature for 30 min. Crystal violet bound to adhered cells (biofilm) was extracted with 200 µL of 30% acetic acid (99.8%; Mikrochem Ltd., Pezinok, Slovak Republic). Absorbance was measured spectrophotometrically at 550 nm. The experiment was conducted in triplicate. The results were expressed as the percentage of biofilm inhibition

% biofilm inhibition = [1 − (A_CFS_/Ao)] × 100

where A_CFS_ is the absorbance of the well content with CFS and Ao is the absorbance of the control well.

#### 2.7.1. Effect of the CFS of Skin Isolates on the Dispersion of Biofilm Produced by *S. aureus* CCM 4223 and MRSA

The effect of the nnCFS and nCFS of *Weissella* spp. and *B. coagulans* on the dispersion of biofilms produced by *S. aureus* CCM 4223 and MRSA was determined according to Kaur et al. [33], with some modifications. Briefly, 25 µL of the standardized suspension of *S. aureus* was added to 2.5 mL of MRS-T. For the preparation of a standardized suspension of *S. aureus*, three to four solitary colonies were resuspended in 5 mL of saline solution and adjusted to the density of one McFarland (approximately 3 × 10^8^ CFU/mL). Subsequently, 200 µL of the sample was pipetted into the wells of 96-well microtiter plates. After incubation at 37 °C for 24 h, the non-adhered cells were removed by gentle pipetting without disrupting the biofilm. A total of 25 µL of nnCFS/nCFS of skin isolates was added to 2.5 mL of MRS-T medium (pH = 7). The pH of nnCFS was in the range of 3.86–4.98. Then, 200 µL of nnCFS and nCFS was added to the biofilm preformed by the pathogenic strain. In the control wells, instead of nnCFS/nCFS, 200 µL of MRS-T medium (pH = 7) was added. The plates were incubated at 37 °C for 48 h. After incubation, the quantification of the biofilm was carried out using a modified crystal violet assay, as described above. The experiment was conducted in triplicate, and the results are presented as the means ± SD.

#### 2.7.2. Effect of Catalase- and Trypsin-Treated nCFS of *Weissella* spp. on the Growth of *S. aureus* CCM 4223

This test was performed according to Wasfi et al. [34], with some modifications. In the nCFS of *Weissella* spp., the effect of hydrogen peroxide was eliminated by treating the supernatant with 0.5 mg/mL of catalase (Sigma-Aldrich, Taufkirchen, Germany), and the effect of potential antimicrobial agents with a proteinaceous nature was eliminated by treating the supernatant with 1 mg/mL of trypsin (Sigma-Aldrich, Taufkirchen, Germany). An aliquot (25 µL) of a standardized suspension of *S. aureus* (approximately 3 × 10^8^ CFU/mL) or sterile saline solution was added to 2.5 mL of the treated and untreated nCFS of *Weissella* spp. Subsequently, a 200 µL sample was pipetted into the wells of 96-well microtiter plates. Trypsin- or catalase-treated MRS-T medium with the saline solution and individual treated nCFS with the saline solution were used as a negative control. MRS-T medium with enzymes with *S. aureus* CCM 4223 without nCFS was used as a positive control. The microtiter plates were incubated aerobically for 24 h at 37 °C. After incubation, the growth of *S. aureus* was measured spectrophotometrically as the optical density at 570 nm. The experiment was conducted in triplicate, and the results are presented as the means ± SD.

### 2.8. Screening of the Ability to Produce Biosurfactants (BS)

#### 2.8.1. Determination of the Extracellular BS

A screening test was used to monitor the production of BS in the CFS of *Weissella* spp. and *B. coagulans* strains. The CFS were obtained as described above. The oil spreading test was performed according to Morikawa et al. [35]. First, 20 mL of distilled water was added to the Petri dish, and 20 μL of crude oil (Slovnaft, Vlčie hrdlo, Bratislava, Slovakia) was dripped onto the water surface, followed by 10 μL of the CFS of the tested strains. MRS–T broth was used as a negative control, and Tween 80 was used as a positive control. The diameter of the clear zones was measured with a ruler in mm, and the diameter of the negative control was subtracted. The experiment was conducted in triplicate, and the results are presented as the means ± SD.

#### 2.8.2. Determination of the Cell-Bound BS

The oil spreading test was performed according to Ghasemi et al. [36]. The pellets obtained during the preparation of CFS were washed twice in demineralized water and resuspended in 20 mL of phosphate-buffered saline (PBS). In order to release BS bound to the cell, the cell-suspension was left at room temperature for 2 h in a roller mixer with gentle shaking (150 rpm). Subsequently, the cells were removed by centrifugation (4173× *g*, 40 min, 4 °C), and the remaining supernatant was tested for the presence of BS. The diameter of the clear zones was measured with a ruler in mm, and the diameter of the negative control was subtracted. The experiment was conducted in triplicate, and the results are presented as the means ± SD.

#### 2.8.3. Screening of the Genes Responsible for the Biosynthesis of the Biosurfactants of *Bacillus coagulans*

Genomic DNA from *Bacillus* spp. was isolated using a High Pure PCR Template Preparation Kit (Roche, Basel, Switzerland). PCR amplification was carried out in 50 µL reaction mixtures containing 2 µL (100 ng) of bacterial DNA, OneTaq 2X Master Mix with standard buffer (New England Biolabs, Foster City, CA, USA), nuclease-free water and primers at a concentration of 33 µM. PCR amplification was performed using a thermocycler (TProfessional Basic, Biometra GmbH, Göttingen, Germany). The primers of the genes used in the PCR amplification were selected according to Chung et al. [37] and Amruta et al. [38] (Table 1). The following parameters were used: initial activation at 95 °C for 3 min; 35 cycles of denaturation at 95 °C for 30 s; annealing for 30 s at variable temperatures depending on the primers used; an extension step at 72 °C for 45 s, except for the *ituC* and *ituD* genes, for which the extension time was 30 and 32 s, respectively. Finally, the amplification was completed by the extension step at 72 °C for 10 min. The surfactin-producing strain *B. subtilis* subsp. *subtilis* DSM 3257 (Leibniz Institute, German Collection of Microorganisms and Cell Cultures GmbH, Braunschweig, Germany) and the fengycin- and iturin-producing strain *B. amyloliquefaciens* 4/9 were used as positive controls. The experiment included a negative control mixture without added DNA. The final product of the amplification reaction was analyzed by electrophoresis using 2% agarose gel and visualized with 2 µL GelRed (Biotium, Inc., Hayward, CA, USA) under UV light. The products obtained from the PCRs were purified and sequenced in forward and reverse directions (Microsynth Austria GmbH, Wien, Austria). The sequences were edited and aligned using Geneious 8.0.5 and analyzed using BLASTn (https://blast.ncbi.nlm.nih.gov/Blast.cgi, accessed on 6 June 2019). The software Sequin (https://www.ncbi.nlm.nih.gov/Sequin/, accessed on 13 June 2019) was used for processing the sequences before submission to the GenBank database. The sequences of the fengycin *fenD* gene of these strains were sent to the GenBank and accepted under Accession number MN197532 for *B. coagulans* 3T27 and MN197533 for *B. coagulans* 9FT27.

### 2.9. Statistical Analysis

The data were analyzed by GraphPad Prism 6.01 software (GraphPad Inc., San Diego, CA, USA). The results were evaluated using an unpaired Student’s *t*-test or Dunnett’s test. The production of organic acids was analyzed by GraphPad Prism 9.4.1 software (GraphPad Inc.), and the results were evaluated by ordinary one-way ANOVA and Tukey’s test. Differences were considered significant at *p* < 0.05.

## 3. Results

### 3.1. Genotypic Identification of Isolates

The results of the 16S rRNA gene sequencing indicated that skin isolates selected on gram-positive staining, non-hemolytic activity, coccoid- or rod-shaped, belonged to the *Weissella* and *Bacillus* genera.

BLASTn analysis showed that the isolates 9FT27 and 3T27 had 99.08% and 99.49% homology with *B. coagulans* NR_041523.1, respectively. The 7/1D23 isolate exhibited 100% similarity with *W. hellenica* NR_113775.1, and the isolate 4/8D37 was 99.72% similar to *W. cibaria* NR_036924.1. *W. hellenica* NR_118771.1 was 99.51% and 99.97% similar to the isolates 4/2D23 and 1/7D23, respectively.

### 3.2. Antibiotic Susceptibility Testing and MIC Determinations

One of the safety considerations in probiotic studies is the verification that a prospective probiotic strain does not contain potentially transferable resistance. The results of the antibiotic susceptibility testing of four *Weissella* spp. isolates and two *B. coagulans* strains are listed in Table 2. The isolate of *B. coagulans* 9FT27 had MIC values for ampicillin and penicillin higher than the microbiological breakpoints; however, certain species of *Bacillus* are typically resistant to penicillin and other β-lactam antibiotics as a result of the production of β-lactamase enzymes [39], and the guidance on the assessment of bacterial antimicrobial susceptibility [27,28] described microbiological cut-off values (mg/L) for ampicillin as not required.

### 3.3. Organic Acids Production Analysis

The results of the organic acid production are presented in Table 3. The main products were lactic, acetic and acetoacetic acid. *W. cibaria* 4/8D37 and *W. hellenica* 4/2D23 showed a higher production of the above-mentioned organic acids compared to *W. hellenica* 1/7D23 and 7/1D23. The production of acetic and acetoacetic acid by *B. coagulans* 9FT27 was higher compared to that by *B. coagulans* 3T27. Formic and succinic acid production ranged up to 10 mM/L in all tested strains.

The production of lactic acid was significantly higher (*p* < 0.01) in *W. hellenica* 4/2D23 than it was in *W. hellenica* 1/7D23 and *W. hellenica* 7/1D23 and significantly higher (*p* < 0.05) than it was in *B. coagulans* 3T27. *W. cibaria* 4/8D37 produced significantly more (*p* < 0.001) lactic acid than *W. hellenica* 1/7D23 and *W. hellenica* 7/1D23 and significantly more (*p* < 0.01) lactic acid than *B. coagulans* 3T27 and *B. coagulans* 9FT27. In *B. coagulans* 3T27, the production of lactic acid was significantly higher (*p* < 0.05) than it was in *W. hellenica* 1/7D23 and *W. hellenica* 7/1D23. The production of lactic acid was significantly higher (*p* < 0.01) in *B. coagulans* 9FT27 than it was in *W. hellenica* 1/7D23 and significantly higher (*p* < 0.05) than it was in *W. hellenica* 7/1D23.

The production of acetic acid was significantly higher (*p* < 0.001) in *W. hellenica* 4/2D23, *W. cibaria* 4/8D37 and *B. coagulans* 9FT27 than it was in *W. hellenica* 1/7D23, *W. hellenica* 7/1D23 and *B. coagulans* 3T27.

The production of acetoacetic acid was significantly higher (*p* < 0.001) in *W. hellenica* 4/2D23 than it was in the rest of the tested strains. In *W. cibaria* 4/8D37 and *B. coagulans* 9FT27, the production of acetoacetic acid was significantly higher (*p* < 0.001) than it was in *W. hellenica* 1/7D23, *W. hellenica* 7/1D23 and *B. coagulans* 3T27.

### 3.4. Effect of the CFS of Skin Isolates on the Growth and Biofilm Formation of S. aureus CCM 4223 and MRSA

**Figure 1:**
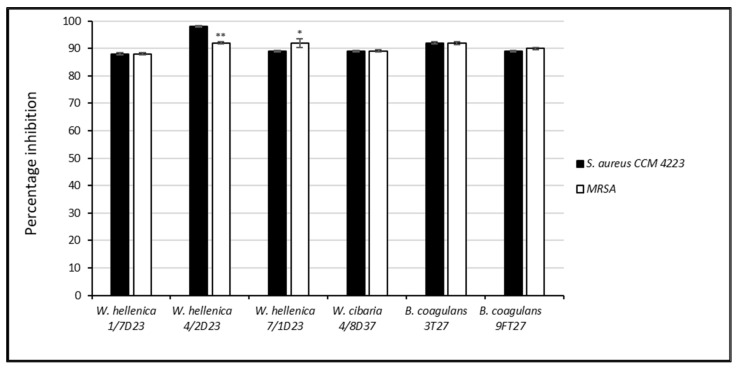
Percentage inhibition of the growth of *Staphylococcus aureus* CCM 4223 and methicillin-resistant *S. aureus* by the nnCFS of skin isolates. The experiment was conducted in triplicate, and the results are presented as the means ± SD. ** *p* < 0.01; * *p* < 0.05.

**Figure 2:**
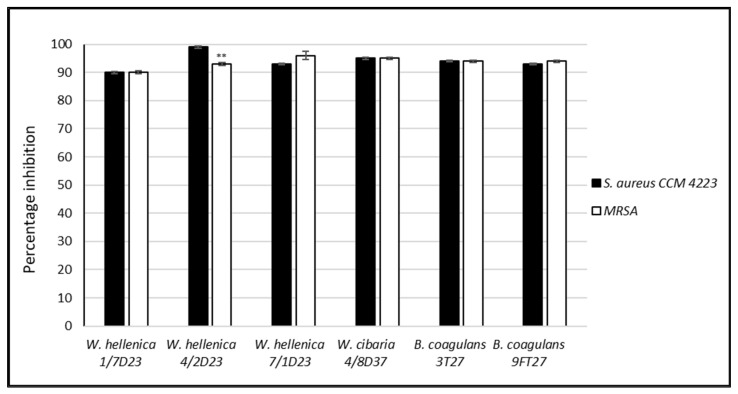
Percentage inhibition of the biofilm of *Staphylococcus aureus* CCM 4223 and methicillin-resistant *S. aureus* by the nnCFS of skin isolates. The experiment was conducted in triplicate, and the results are presented as the means ± SD. ** *p* < 0.01.

The nnCFS of all tested isolates inhibited the growth and biofilm formation of *S. aureus* CCM 4223 and MRSA in the range of 88‒99% (Figure 1 and Figure 2). A significantly lesser (*p* < 0.01) growth inhibition of MRSA was exhibited by *W. hellenica* 4/2 D23 compared to *S. aureus* CCM 4223. A significantly higher (*p* < 0.05) growth inhibition of MRSA was exhibited by *W. hellenica* 7/1 D23 compared to *S. aureus* CCM 4223.

*W. hellenica* 4/2 D23 showed a significantly lesser (*p* < 0.01) inhibition of the biofilm formation of MRSA than *S. aureus* CCM 4223.

**Figure 3:**
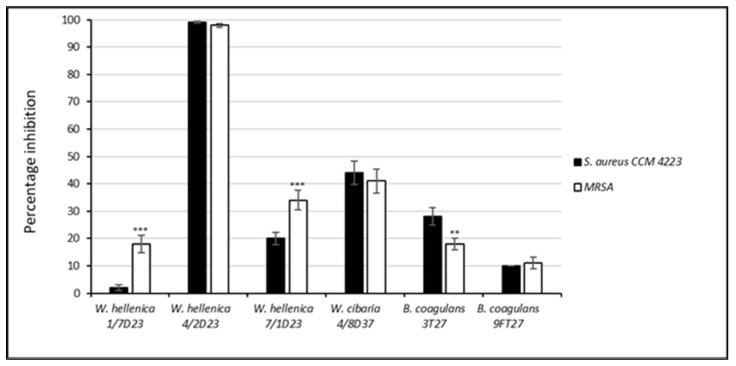
Percentage inhibition of the growth of *Staphylococcus aureus* CCM 4223 and methicillin-resistant *S. aureus* by the nCFS of skin isolates. The experiment was conducted in triplicate, and the results are presented as the means ± SD. *** *p* < 0.001; ** *p* < 0.01.

**Figure 4:**
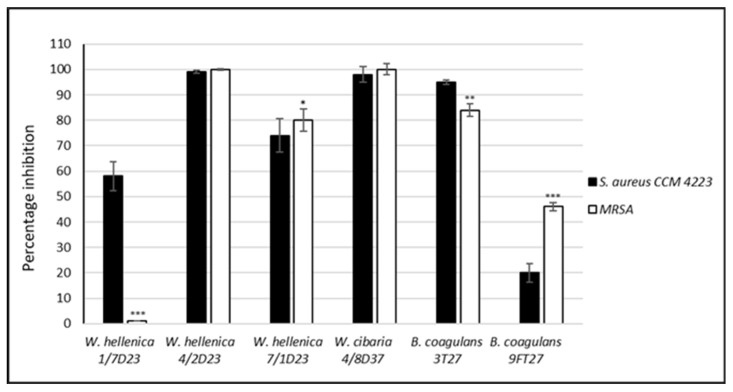
Percentage inhibition of the biofilm of *S. aureus* CCM 4223 and MRSA by the nCFS of skin isolates. The experiment was conducted in triplicate, and the results are presented as the means ± SD. *** *p* < 0.001; ** *p* < 0.01; * *p* < 0.05.

The results of the antibacterial and antibiofilm activity of the nCSF of *Weissella* spp. and *B. coagulans* against *S. aureus* CCM 4223 and MRSA are shown in Figure 3 and Figure 4. The neutralized supernatant of *W. hellenica* 1/7D23 inhibited the growth of MRSA in 18% but did not inhibit the growth of *S. aureus* CCM 4223. Nearly 100% growth inhibition of both pathogenic strains was exhibited by the nCFS of *W. hellenica* 4/2D23. *W. cibaria* 4/8D37 inhibited the growth of MRSA and *S. aureus* CCM 4223 nearly equally (44% and 41%, respectively). The lowest growth inhibition of the MRSA was accomplished by the nCFS of *B. coagulans* 9FT27 (11%). The nCFS of *B. coagulans* 3T27 inhibited the growth of MRSA by 18% and the growth of *S. aureus* CCM 4223 by 28%. A significantly higher (*p* < 0.001) growth inhibition of MRSA was exhibited by *W. hellenica* 1/7D23 and *W. hellenica* 7/1D23 compared to *S. aureus* CCM 4223. The nCFS of *B. coagulans* 3T27 inhibited the growth of MRSA significantly less (*p* < 0.01) than the growth of *S. aureus* CCM 4223.

The neutralized supernatant of *W. hellenica* 1/7D23 did not inhibit the biofilm formation of MRSA but inhibited the biofilm formation of *S. aureus* CCM 4223 by 58%. The nCFS of *W. hellenica* 4/2D23 completely inhibited the biofilm formation of both pathogenic strains. The nCFS of *W. hellenica* 7/1D23 and *W. cibaria* 4/8D37 inhibited the biofilm formation of MRSA by 80% and 100% and the biofilm formation of *S. aureus* CCM 4223 by 74% and 98%. The nCFS of *B. coagulans* 3T27 and 9FT27 inhibited the biofilm formation of MRSA by 84% and 46% and the biofilm formation of *S. aureus* CCM 4223 by 95% and 20%, respectively. *W. hellenica* 7/1D23 and *B. coagulans* 9FT27 inhibited significantly more (*p* < 0.05 and *p* < 0.001, respectively) of the biofilm formation of MRSA than *S. aureus* CCM 4223. On the contrary, *W. hellenica* 1/7D23 and *B. coagulans* 3T27 inhibited significantly less (*p* < 0.001 and *p* < 0.01, respectively) of the biofilm formation of MRSA than *S. aureus* CCM 4223.

### 3.5. Effect of the CSF of Skin Isolates on the Dispersion of Biofilm Produced by S. aureus CCM 4223 and MRSA

**Figure 5:**
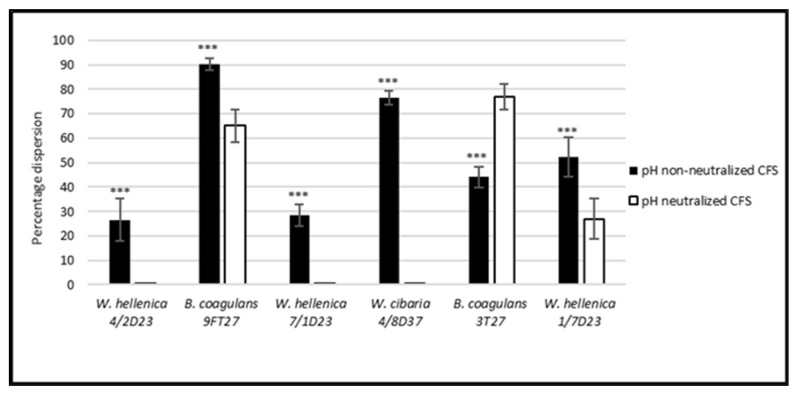
Percentage dispersion of the preformed biofilm of *S. aureus* CCM 4223 by the CFS of skin isolates. The experiment was conducted in triplicate, and the results are presented as the means ± SD. *** *p* < 0.001.

**Figure 6:**
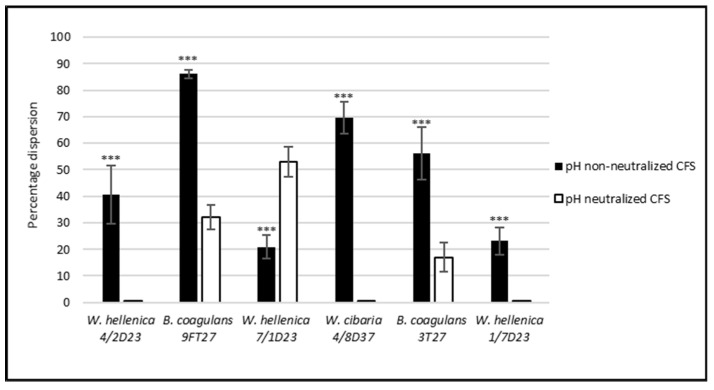
Percentage dispersion of the preformed biofilm of MRSA by the CFS of skin isolates. The experiment was conducted in triplicate, and the results are presented as the means ± SD. *** *p* < 0.001.

The results obtained by the investigation of the dispersion activity of the nnCFS and nCSF of *Weissella* spp. and *B. coagulans* on 24 h biofilms preformed by *S. aureus* CCM 4223 and MRSA are shown in Figure 5 and Figure 6. All of the tested nnCFS of the skin isolates exhibited dispersion activity as they disrupted the preformed biofilm of *S. aureus* CCM 4223 in the range of 26 to 90%. The dispersion activity of nCFS was observed in *W. hellenica* 1/7D23 and in both strains of *B. coagulans* (9FT27, 3T27), which disrupted the biofilm produced by the pathogenic strain in the range of 27–77%. The dispersion activity of nnCFS was significantly higher (*p* < 0.001) than that of nCFS, except for the nCFS of *B. coagulans* 3T27.

The dispersion activity of nnCFS was observed in all of the tested nnCFS of the skin isolates, which disrupted the biofilm produced by MRSA in the range of 21–86%. The dispersion activity of nCFS was observed in *W. hellenica* 7/1D23 and in both strains of *B. coagulans* (9FT27, 3T27), which disrupted the biofilm produced by MRSA in the range of 17–53%. The dispersion activity was significantly higher (*p* < 0.001) in nnCFS than it was in nCFS, except for the nnCFS of *W. hellenica* 7/1D23.

**Figure 7:**
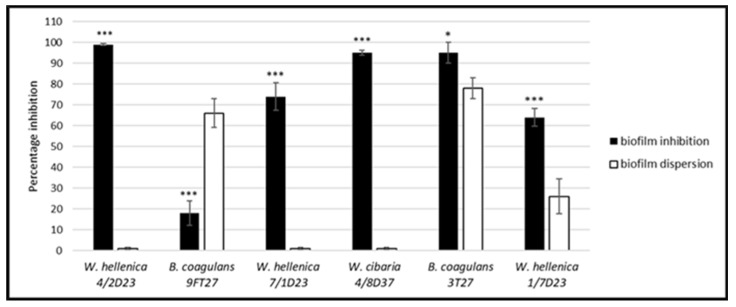
Comparison of the effect of the skin isolates’ nCFS on the biofilm formation and dispersion of the 24 h-old preformed biofilm of *S. aureus* CCM 4223. The experiment was conducted in triplicate, and the results are presented as the means ± SD. *** *p* < 0.001; * *p* < 0.05.

A comparison of the effect of the nCFS of skin isolates on the biofilm formation and dispersion of 24 h biofilms preformed by *S. aureus* CCM 4223 (Figure 7) showed that the effect of the nCFS of all *Weissella* spp. and *B. coagulans* 3T27 on biofilm inhibition was significantly (*p* < 0.001; *p* < 0.05) higher than their dispersion activity. On the contrary, the nCFS of *B. coagulans* 9FT27 showed significantly (*p* < 0.001) lower biofilm inhibition in comparison with dispersion activity.

**Figure 8:**
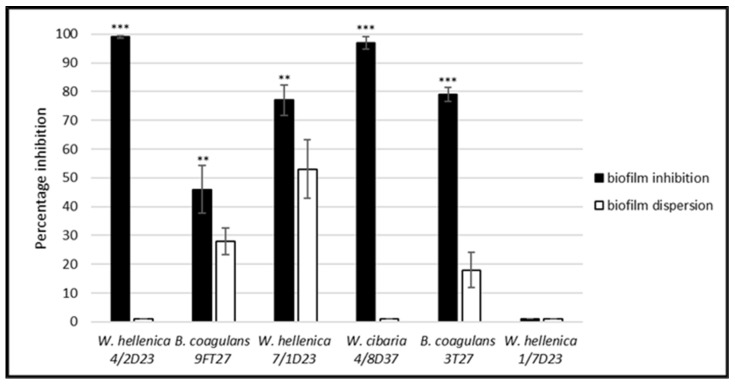
Comparison of the effect of the nCFS of skin isolates on the biofilm formation and dispersion of 24 h-old preformed biofilms of MRSA. The experiment was conducted in triplicate, and the results are presented as the means ± SD. *** *p* < 0.001; ** *p* < 0.01.

A comparison of the effect of the nCFS of skin isolates on the biofilm formation and dispersion of the 24 h-old preformed biofilms of MRSA (Figure 8) showed that the effect of the nCFS of *W. hellenica* 4/2D23 (*p* < 0.001), *B. coagulans* 9FT27 (*p* < 0.01), *W. hellenica* 7/1D23 (*p* < 0.01), *W. cibaria* 4/8D37 (*p* < 0.001) and *B. coagulans* 3T27 (*p* < 0.001) on the biofilm inhibition was significantly higher than their dispersion activity.

### 3.6. Antimicrobial Activity of the Treated nCFS of Weissella spp.

**Figure 9:**
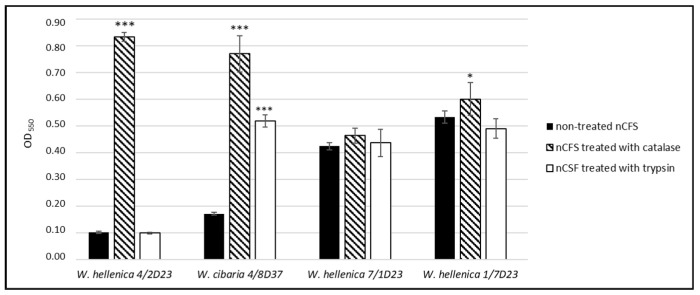
*S. aureus* CCM 4223’s growth, expressed in optical density at 550 nm, in the presence of treated and untreated *Weissella* spp. nCFS. The experiment was conducted in triplicate, and the results are presented as the means ± SD. They were ignificantly different compared with the non-treated nCFS (*** *p* < 0.001; * *p* < 0.05).

The effect of the nCFS of *W. hellenica* 4/2D23 on the growth of *S. aureus* CCM 4223 (Figure 9) was significantly (*p* < 0.001) negated by the catalase treatment when compared to the untreated nCFS. This suggested that the inhibition activity could be attributed to the production of hydrogen peroxide. The inhibition activity of the nCFS of *W. cibaria* 4/8D37 was significantly (*p* < 0.001) negated by both treatments (catalase and trypsin) when compared to the untreated supernatant, indicating the role of hydrogen peroxide and the antimicrobial protein in the inhibition activity. There was no significant difference in the inhibition of the growth of *S. aureus* CCM 4223 between the treated and untreated nCFS of *W. hellenica* 7/1D23. After neutralizing the supernatant acidity, the antimicrobial effect of the CFS of *W. hellenica* 1/7D23 was slightly but significantly (*p* < 0.05) negated by the catalase treatment in comparison with the untreated supernatant.

### 3.7. Screening of the Ability to Produce Biosurfactants (BS)

The results (Table 4) showed that *Weissella* spp. produced only extracellular BS, whereas *B. coagulans* 3T27 produced cell-bound BS and *B. coagulans* 9FT27 produced both types of BS.

### 3.8. Screening of the Genes Responsible for the Production of BS in B. coagulans

PCR screening assays for the genes (surfactin *sfp*, surfactin *srfAA*, fengycin *fenB* and iturin *ituC*) encoding the production of BS in *Bacillus* spp. determined the presence of the fengycin *fenD* product (269 bp) in *B. coagulans* 3T27 and *B. coagulans* 9FT27. Based on the sequence alignment analysis, the percentage identity of the fengycin *fenD* gene in *B. coagulans* 3T27 and *B. coagulans* 9FT27 was 95%.

Based on the sequencing of the amplified *fenD B. coagulans* gene, it was confirmed that the *fenD* primers used in the study were highly gene-specific. On the other hand, the primers were designed for *fenD B. amyloliquefaciens*, and, therefore, they were not subspecies-specific.

## 4. Discussion

Antimicrobial resistance poses a serious global threat and is becoming a growing concern due to its potential impact on humans as well as animals [40]. Since it seems that the occurrence and transmission of antibiotic-resistant microorganisms increase due to the more frequent topical application of antibiotics rather than their systemic use, the search for alternatives, e.g., probiotic microorganisms and their bioactive substances, is necessary [41,42]. When skin dysbiosis occurs as a result of topical antibiotic therapy, probiotics can act as modulators and restore the microbial balance [4,43]. *S. aureus* is a pathogen causing several skin infections as well as other systemic infections, and the importance of biofilms in its pathogenicity has been described [44,45].

In the present study, we focused on the isolation of the potentially beneficial bacteria from the skin of various equine body regions and the screening of their antimicrobial and antibiofilm activity against biofilm-forming *S. aureus* strains. The isolates were identified, based on the consensus sequence of the 16S rRNA gene and genotyping, as *W. cibaria*, *W. hellenica* and *B. coagulans*.

*Weissella* spp. are mainly used in the food industry in the production of fermented foods and as probiotics [14,46]. It was reported that *W. hellenica* DS-12 was found to be suitable for aquaculture due to its antimicrobial activity against fish pathogens, e.g., *Edwardsiella*, *Pasteurella*, *Aeromonas* and *Vibrio* [47]. *W. cibaria* CMS1, which inhibited *Str. mutans*, is known for preventing biofilm formation in the oral cavity [13], and *W. cibaria* JW15 is a probiotic feed additive for dogs [48]. To the best of our knowledge, there are no reports regarding the skin application of *Weissella* spp. in humans or animals.

*B. coagulans* is a well-established probiotic within food formulations due to its high resistance to degradation [49]. In addition, it has been shown to have beneficial effects for the host microbiome [50], metabolism [51] and immunity [52].

In general, *Weissella* spp. are not considered as Generally Recognized As Safe (GRAS) and thus could not be used as probiotics without restrictions. Studies on the antibiotic resistance profiles of the *Weissella* genus are very limited, and MIC breakpoints have not been defined by the EFSA. The MICs of *Weissella* strains were compared to the epidemiological cut-off values reported by Flórez et al. [53] and defined according to the EFSA [27] for the genera *Leuconostoc* because of their phylogenetic relationship [54]. The MIC values of 16 antibiotics encompassing nearly all important pharmaco-pharmacological classes were determined for our strains of *Weissella* spp. by the broth microdilution method in VetMIC plates. Our results showed that the antibiotic resistance of these strains was found to be equal to or lower than the cut-off value. *W. hellenica* and *W. cibaria* were not susceptible to vancomycin (MICs ≥ 128 μg/mL and MICs ≥ 16 μg/mL, respectively), since this is a common trait for species belonging to the *Leuconostoc*-*Weissella* group [55]. Such intrinsic characteristic is linked to the presence of D-Ala-D-Lactate in their peptidoglycan rather than to the D-Ala-D-Ala dipeptide [56].

*B. coagulans* has been granted GRAS status by the US Food and Drug Administration [57]. The testing of the MICs of *B. coagulans* showed that both strains were sensitive to all tested antibiotics except for *B. coagulans* 9FT27, which had MIC values for ampicillin and penicillin that were higher than the microbiological breakpoints. However, certain species of *Bacillus* are considered inherently resistant to β-lactam antibiotics, and the guidance on the assessment of bacterial antimicrobial susceptibility [27,28] described the microbiological cut-off values (mg/L) for ampicillin and penicillin as not required. The isolates of *Weissella* spp. and *B. coagulans* did not exhibit hemolytic activity.

The present study focused on the effect of the CFS obtained from *Weissella* spp. and *B. coagulans* on the growth and biofilm formation of the pathogenic strains *S. aureus* CCM 4223 and MRSA. Our results showed that the growth of *S. aureus* CCM 4223 and MRSA was nearly completely inhibited by nnCFS (pH < 4.5). This inhibitory effect was mainly due to the production of organic acids, as their neutralization resulted in a higher-than-60% decrease in the percentage of inhibition from the initial level, except for the CFS of *W. hellenica* 4/2D23 (no decrease in growth inhibition) and *W. cibaria* 4/8D37 (a decrease in growth inhibition lower than 50%). Upon fermentation, the pH of the nnCFS was reduced to less than 4.5 for all skin isolates, which was unfavorable for the growth of *S. aureus*, as its survival requires the pH to be in the range of 4.5–9.3 [58,59]. In this study, the capillary isotachophoretic analysis of the CFS of *Weissella* spp. and *B. coagulans* confirmed the presence of a considerable amount of lactic, acetic and acetoacetic acids. The antimicrobial effect of organic acids is associated with the disruption of the membrane potential of the attacked cells, the inhibition of active transport across the membrane, the reduction in intracellular pH and the inhibition of various metabolic functions of the bacterial cell [60]. Organic acids are generally more effective (at the same pH) than inorganic acids at preventing bacterial growth [61]. This has led to their extensive use as preservatives in the food industry [62] and also to their increased use as topical agents for the treatment of infected wounds [63,64]. *B. coagulans* and *Weissella* spp. are important producers of lactic acid and acetic acid [65,66]. Based on the strong inhibition of the growth of *S*. Typhi DMST 22842, organic acids (lactic acid and acetic acid) were the main antibacterial metabolites found in the CFS of the strain *Weissella confusa* WM36 [67]. The supernatants of *B. coagulans* MTCC 5856, *B. coagulans* GBI-30 and *B. coagulans* T242 exerted a broad spectrum of antimicrobial activity, resulting in the inhibition of the growth of gram-positive (including *S. aureus*) and gram-negative pathogens [68,69,70].

The antibiofilm effect of the nnCFS (pH < 4.5) of *Weissella* spp. and *B. coagulans* was related to their antimicrobial effect on the growth of the pathogenic strain. Our results showed that the pH-neutralized CFS of *W. hellenica* 4/2D23 and *W. cibaria* 4/8D37 did not reduce the percentage of the inhibition of the biofilm formation in *S. aureus*. The partial inhibition (15–30%) of *S. aureus* biofilm formation by the nCFS from the *W. hellenica* strains 1/7D23 and 7/1D23 was detected. This suggested that antimicrobial or antibiofilm compounds other than organic acids may be involved in the inhibition of biofilm formation.

The catalase or trypsin treatment of the pH-neutralized CFS of *W. hellenica* 4/2D23, *W. cibaria* 4/8D37 and *W. hellenica* 1/7D23 significantly altered the growth inhibition of *S. aureus* CCM 4223, thereby showing that hydrogen peroxide and/or the protein bioactive compound (bacteriocin) could be responsible for their antimicrobial activity. However, this suggestion needs to be confirmed by the isolation/detection and characterization of these compounds.

Bacteriocins such as weissellicin Y, M and L were detected in *W. hellenica* QU 13 and *W. hellenica* 4–7, which showed a broad inhibitory spectrum against gram-positive bacteria [71,72]. Weissellicin 110 was detected in *Weissella cibaria* 110 isolated from the Thai fermented fish product plaa-som; however, it showed only a narrow spectrum of inhibition of other lactobacilli [73]. On the other hand, weissellicin D determined in *W. hellenica* exhibited a broad range of antimicrobial activity against food-borne spoilage and pathogenic bacteria, such as *S. aureus*, *L. monocytogenes* and *E. coli*, as well as against some yeasts and molds [74]. Six strains of *W. cibaria* produced large amounts (2.2–3.2 mM/L) of hydrogen peroxide in supplemented media [75]. Moreover, it was reported that *Weissella kimchii* PL9023 inhibited the growth and adherence of vaginal isolates of *C. albicans*, *E. coli*, *S. aureus* and *Streptococcus agalactiae* by hydrogen peroxide production [76]. In addition to organic acids, *B. coagulans* can also secrete other antimicrobial substances such as bacteriocins. *B. coagulans* I_4_ was the first strain of *B. coagulans* that was found to produce a bacteriocin-like inhibitory substance (BLIS) named coagulin [77], which is a broad-spectrum antibacterial agent that is effective against gram-positive bacteria [10,78]. In our study, the exclusion of the inhibitory effect of organic acids by the neutralization of the CFS of *B. coagulans* 3T27 showed a nearly 100% inhibition of biofilm formation by *S. aureus*. This suggested the production of bacteriocin; however, further experiments are needed to confirm this finding.

Our results showed that the percentage of dispersion activity was higher in the nnCFS of *Weissella* spp., while in *B. coagulans* strains, the disruption of the biofilm preformed by *S. aureus* was similar to both of the tested CFS. The assumption is that the production of organic acids (lactic and acetic acid) and/or H_2_O_2_ was responsible for the dispersion activity of the acidic CFS of *Weissella* spp., but in the case of *B. coagulans* isolates, this may be a combined effect of organic acids and the production of a compound with dispersion activity, such as fengycin lipopeptide. Tsang et al. [79] found that a clinically acceptable concentration of acetic acid (5%) eradicated more than 96% of the 24 h preformed biofilm of methicillin-sensitive *S. aureus* (MSSA) within a 20 min treatment time. The pH of the cytoplasm is reduced by the intracellular dissociation of acetic acid, resulting in protein unfolding and the subsequent damage to both the membrane and DNA [80].

Bjarnsholt et al. [63] also reported the complete eradication of the three-day-old biofilm of *S. aureus* and *P. aeruginosa* by treatment with 1% acetic acid. The maximum antibacterial effect of acetic acid was observed at pH 4.76, which was also the dissociation equilibrium point of acetic acid. Moreover, the authors found that the usage of physiologically tolerable concentrations of acetic acid promoted the nontoxic treatment of biofilms in chronic hard-to-heal wounds. In our study, acidic CFS had a pH < 4.5. When the effect of a low pH was eliminated by neutralization, the percentage of preformed biofilm dispersion was significantly lowered. Peetermans et al. [81] reported a synergistic effect of lactic and acetic acid on the growth of *Saccharomyces cerevisiae*. It was supposed that lactic acid decreased the pH of the medium, thereby increasing the toxicity of the non-dissociated fraction of acetic acid [82]. It was recommended to lower the pH of the solution to maximize the availability of the non-dissociated acetic acid and its antibacterial effect [61]. Uppuluri et al. [83] found that the pH of the growing medium exerts an important effect on *C*. *albicans* biofilm dispersion, which is enhanced at an acidic pH and decreases under alkaline conditions.

Unsaturated fatty acids are another example of organic acids that exhibit broad-spectrum biofilm-detaching activity in vitro. According to Davies and Marques [84] and Rahmani-Badi et al. [85], a short-chain fatty acid signaling molecule, cis-2-decenoic acid, which is produced by *P*. *aeruginosa*, was found to disperse *Klebsiella pneumoniae*, *E*. *coli*, *Bacillus subtilis*, *S*. *aureus*, *Str. mutans*, *Streptococcus pyogenes* and even *Candida* biofilms in competition experiments. This observation has led to the hypothesis that biofilm dispersion may result from the accumulation of extracellular messenger cis-monounsaturated fatty acids, which act as an inducer of the release of cells from the biofilm.

Another substance that affects biofilm dispersion is H_2_O_2_. As mentioned before, the catalase treatment of the pH-neutralized CFS of our strains of *Weissella* spp. significantly inhibited the growth of *S. aureus* CCM 4223, which indicated the possibility of H_2_O_2_ production.

Lipopeptides are one of the largest groups of BS that can effectively disperse microbial biofilms [86]. Lactic acid bacteria BS are generally produced either as cell-bound compounds or as excreted compounds [87]. Subsanguan et al. [16] reported both ways of BS production by immobilized *W. cibaria* PN3 cells. According to the oil spreading test, our *Weissella* strains produced only extracellular BS. The extracellular BS production in *B. coagulans* was determined in a study by Huszcza and Burczyk [88]. Extracellular and cell-bound BS were produced by *B. tequilensis* [89]. In our study, *B. coagulans* 3T27 produced cell-bound BS, and *B. coagulans* 9FT27 produced both types of BS. Moreover, the presence of the *fenD* gene, associated with biosurfactants production, was detected in *B. coagulans* for the first time. It was described previously in other *Bacillus* strains, e.g., *B. subtilis* [90], *B. pumilus* [91], *B*. *mojavensis* [92] and *B. amyloliquefaciens* [31]. Fengycin-like lipopeptides are derived from *B. subtilis* and *Bacillus licheniformis* and are involved in the inhibition of biofilms [93], causing up to 90% dispersion of gram-positive *S. aureus* biofilms and up to 97% dispersion of gram-negative *E. coli* biofilm [94]. Our results suggested the effect of fengycin on biofilm dispersion, but one needs to test this effect when using isolated and purified compounds.

## 5. Conclusions

Our investigations showed that six finally selected strains of *Weissella* spp. and *B. coagulans* may have a potential application in reducing *S. aureus* in equine dermatitis. According to in vitro tests, the most suitable candidate for a skin inoculant seems to be the strain of *W. cibaria* Biocenol™ 4/8D37 CCM 9015. The strain produces organic acids, mainly lactic and acetic acid, is susceptible to antibiotics and has antimicrobial, antibiofilm and dispersion activity against the biofilm-forming strains *S. aureus* CCM 4223 and MRSA. The effect of the topically applied strain *W. cibaria* Biocenol™ 4/8D37 CCM 9015 on the microbiota of the affected skin requires further investigation using animal models.

## 6. Patents

Patent application: PP 50058-2021: Microbial strain *Weissella cibaria* 4/8 D37 CCM 9015 bacterial culture, cell-free supernatant of the strain and the pharmaceutical composition containing this. Filed 12 November 2021, Industrial Property Office of the Slovak Republic, Banská Bystrica.

## Figures and Tables

**Table 1:** The primers used for the screening of the genes responsible for biosurfactant biosynthesis.

Gene	Sequence	PCR Product [bp]	Annealing Temperature [°C]
surfactin *sfp*	F-5′-ATGAAGATTTACGGAATTTA-3′ R-5′-TTATAAAAGCTCTTCGTACG-3′	675	50
*srfAA*	F-5′-TCGGGACAGGAAGACATCAT-3′ R-5′-CCACTCAAACGGATAATCCTGA-3′	201	60
fengycin *fenB*	F-5′-CCTGGAGAAAGAATATACCGTACCY-3′ R-5′-GCTGGTTCAGTTKGATCACAT-3′	670	57
*fenD*	F-5′-GCTGGTTCAGTTKGATCACAT-3′ R-5′-GTCATGCTGACGAGAGCAAA-3′	269	61
iturin *ituD*	F-5′-TTGAAYGTCAGYGCSCCTTT-3′ R-5′-TGCGMAAATAATGGSGTCGT-3′	482	57
*ituC*	F-5′-GGCTGCTGCAGATGCTTTAT-3′ R-5′-TCGCAGATAATCGCAGTGAG-3′	423	60

**Table 2:** Minimum inhibitory concentration (MIC-μg/mL) values of the skin isolates.

ATB	*W. hellenica* 1/7 D23	*W. hellenica* 4/2 D23	*W. hellenica* 7/1 D23	*W. cibaria* 4/8 D37	*B. coagulans* 3T27	*B. coagulans* 9FT27
Gm	0.5	0.5	0.5	0.5	0.5	0.5
Km	2	4	2	8	2	2
Sm	4	16	16	16	0.5	0.5
Nm	0.5	2	1	2	0.5	0.5
Tc	4	4	4	4	0.5	0.5
Em	0.12	0.12	0.25	0.5	0.5	0.12
Cl	0.03	0.5	0.03	0.25	0.12	0.12
Cm	2	4	2	2	4	8
Am	0.06	2	2	0.25	0.12	**16**
Pc	0.12	1	1	1	0.06	**16**
Va	**128**	**128**	**128**	**16**	0.25	0.5
Vi	0.5	1	0.5	1	1	0.5
Lz	1	1	1	2	1	2
Tm	2	8	8	8	0.25	1
Ci	8	4	8	16	0.25	0.5
Ri	2	2	4	4	0.12	1

The minimum inhibitory concentration (MIC) of several antibiotics was determined using VetMIC (National Veterinary Institute of Sweden, Uppsala, Sweden) plates, containing serial two-fold dilutions of 16 antibiotics (Gm—gentamicin, Km—kanamycin, Sm—streptomycin, Nm—neomycin, Tc—tetracycline, Em—erythromycin, Cl—clindamycin, Cm—chloramphenicol, Am—ampicillin, Pc—penicillin, Va—vancomycin, Vi—virginiamycin, Lz—linezolid, Tm—trimethoprim, Ci—ciprofloxacin and Ri—rifampicin); bold letters—certain species that are inherently resistant. *W. hellenica*—*Weissella hellenica*, *W. cibaria*—*Weissella cibaria*, *B. coagulans*—*Bacillus coagulans.*

**Table 3:** Analysis of organic acids in the CFS of *Weissella* spp. and *Bacillus coagulans* (mM/L).

Strain	Acid				
	Formic	Lactic	Acetic	Succinic	Acetoacetic
*W. hellenica* 1/7D23	1.68 ± 0.38	14.93 ± 0.80	25.13 ± 1.66	7.41 ± 0.99	8.82 ± 0.33
*W. hellenica* 4/2D23	5.70 ± 0.52	88.69 ± 1.28 **^,^*	101.95 ± 3.19 ***	8.25 ± 0.23	47.75 ± 2.73 ***
*W. hellenica* 7/1D23	2.41 ± 0.37	20.63 ± 0.71	25.67 ± 1.66	6.84 ± 0.57	8.28 ± 0.33
*W. cibaria* 4/8D37	2.08 ± 1.88	119.77 ± 2.47 ***^,^**	96.61 ± 3.59 ***	8.69 ± 0.52	35.07 ± 1.66 ***
*B. coagulans* 3T27	6.61 ± 3.84	52.75 ± 15.75 *	21.07 ± 1.99	8.05 ± 0.19	9.36 ± 0.04
*B. coagulans* 9FT27	6.78 ± 1.66	61.15 ± 4.74 **^,^*	84.02 ± 4.11 ***	7.55 ± 0.18	37.82 ± 2.01 ***

The results are presented as the means ± SD. *** *p* < 0.001; ** *p* < 0.01; * *p* < 0.05.

**Table 4:** The diameter of clear zones in mm.

Strains	Extracellular BS	Cell-Bound BS
*W. hellenica* 1/7D23	20 ± 0.2	negative
*W. hellenica* 4/2D23	8 ± 0.1	negative
*W. hellenica* 7/1D23	8 ± 0.1	negative
*W. cibaria* 4/8D37	16 ± 0.2	negative
*B. coagulans* 3T27	negative	15 ± 0.2
*B. coagulans* 9FT27	8 ± 0.1	12 ± 0.1

The experiment was conducted in triplicate, and the results are presented as the means ± SD.

## Data Availability

The data presented in this study are available on request from the corresponding author.
